# ﻿A review of Gryllidae (Grylloidea) with the description of one new species and four new distribution records from the Sindh Province, Pakistan

**DOI:** 10.3897/zookeys.1078.69850

**Published:** 2021-12-15

**Authors:** Riffat Sultana, Surriya Sanam, Santosh Kumar, Sheik Mohammad Shamsudeen R, Fakhra Soomro

**Affiliations:** 1 Department of Zoology, University of Sindh, Jamshoro, Sindh, Pakistan University of Sindh Jamshoro Pakistan; 2 Department of Zoology, Cholistan University of Veterinary and Animal Sciences, Bahawalpur, Punjab, Pakistan Cholistan University of Veterinary and Animal Sciences Bahawalpur Pakistan; 3 Department of Zoology, Sir Syed college, Kannur University, Kerala, India Kannur University Kerala India; 4 Department of Zoology, Shah Abdul Latif University, Khairpur, Sindh, Pakistan Shah Abdul Latif University Khairpur Pakistan

**Keywords:** *Acheta*, *Callogryllus*, *Miogryllus*, *Modicogryllus*, new distribution record, review, taxonomic key

## Abstract

Seventeen species of the family Gryllidae were reviewed and a *Modicogryllussindhensis* is described herein as new. Four species, namely *Achetahispanicus* Rambur, 1838, *Gryllusseptentrionalis* F. Walker, 1869, *Callogryllussaeedi* Saeed, 2000, and *Miogryllusitaquiensis* Orsini & Zefa, 2017 are recorded as new country and state records. Differences between similar species and a taxonomic key to the species of Sindh are provided.

## ﻿Introduction

Crickets are representative of superfamily Grylloidea with six (four families: Myrmecophilidae, Gryllotalpidae, Mogoplistidae and Gryllidae) Baissogryllidae Gorochov, 1985, Gryllidae Laicharting, 1781, Mogoplistidae Costa, 1855, Phalangopsidae Blanchard, 1845, Protogryllidae Zeuner, 1937 and Trigonidiidae Saussure, 1874 (Cigliano 2021). The group dates back from the Triassic Period and today includes 3,700 for all species of orthopterans known living and 43 extinct species, 22 extant and 27 extinct subfamilies, and 528 extant and 27 extinct genera (Resh and Carde 2009). The Orthoptera Species File is a taxonomic database of the world’s Orthoptera including grasshoppers, katydids, crickets, and related insects, both living and fossil. It has full taxonomic and synonymic information for more than 29,060 valid species and includes 47,500 scientific names and 106,200 specimen records.

Crickets live in virtually all terrestrial habitats from treetops to a meter or more beneath the ground. Field crickets live in oligotrophic, dry, barren habitats. Crickets are abundantly found at night but conceal themselves in thick vegetation, leaf litter, and under stones and rocks. Crickets are drab, or brightly and intensely coloured. Crickets have an incomplete metamorphosis with three life stages viz., egg, nymph, and adults. Females insert their eggs in soil and lay their egg on plants (Alexander 1962).

The classification of the Gryllidae has been established by Henri de Saussure in a remarkable monograph published in Geneva in the years 1877 and 1878. In this thorough work, the author points out the most important morphological characters and establishes the larger divisions of the group. Although a great number of species have been described since the publication of Saussure’s work, it remains the basis of the modern classification of the Grylloidea. The Gryllidae are abundant throughout Sindh, the most cultivated region of Pakistan that are damaged by mole crickets, ground crickets, field crickets, house crickets, etc. The Gryllidae live in different types of habitats such as moist soil, herbs, shrubs, grasses, and vegetation. The fauna of Gryllidae from Sindh is insufficiently known. It was therefore felt necessary to revise the family from this region. Descriptions, taxonomic keys, and illustrations for all 17 known species are provided; bionomics and ecological accounts are also briefly discussed. In this manuscript we offer one new species and four new records from Pakistan, which aid in filling the gaps in our knowledge of the Gryllidae of Pakistan and bring information up to date.

## ﻿Materials and methods

All specimens were collected from different agricultural crops in various districts of Sindh. Material was brought to Entomology and Bio-control Research Laboratory (**EBCRL**), Department of Zoology, University of Sindh, Jamshoro. Methodology for euthanasia was adapted from Vickery and Kevan (1983) and Riffat and Wagan (2015) with slight modifications: specimens were killed by using potassium cyanide or chloroform in standard entomological killing bottles for 5–10 minutes. Samples were not left longer because their colours could change.

Pinning of samples was done quickly after killing. An insect pin was inserted on the pronotum posterior to transverse sulcus, slightly to the right of the median carina. The head was directed slightly downwards on the stretching board. The left wings were set with the long axis of the body nearly at a right angle to the pin. The posterior legs were bent beneath the body to minimize the possibility of breakage and to occupy a smaller area. The abdomen was dropped below the wings and not obscured by the hind legs.

Fully dried specimens were preserved in insect cabinets with labels providing collection date, habitat, locality, and collector’s name. Naphthalene balls (C_10_H_8_) were placed in boxes to prevent the attack of ants and other insects. Specimens were identified through the bibliographies given by Riffat and Wagan (2015), and Orthoptera Species File (**OSF**) (Cigliano et al. 2020) was consulted.

Photographs of the various species were prepared. Line drawings were made with a camera lucida fitted on a microscope (Ernst Leitz Wetzlar Germany 545187) and these were improved with the help of the softwares Adobe illustrator CC-2015 and Adobe Photoshop CS.

Measurements of various body parts were calculated in millimetres (mm) using the microscope (Oculas), 10 × 10 graph, compass, divider, and ruler. Abbreviations used in the text are as follows.

**LH** Length of head;

**LF** Length of femur;

**LP** Length of pronotum;

**LT** Length of tegmen;

**LT** Length of tibia;

**LT** length of tarsus;

**TBL** total body length;

**TN** Tag Number;

**SEMJ** Sindh Entomological Museum Jamshoro.

Species distributions were mapped using latitude and longitude information for available sites of species. The material (TN: 802 SEM) has been deposited in Sindh Entomological Museum Jamshoro (**SEMJ**), Department of Zoology, University of Sindh, Jamshoro. Pakistan.

## ﻿Taxonomic account

### ﻿Family Gryllidae

**Subfamily Gryllinae**

**Tribe Gryllini**

#### Genus *Acheta* Linnaeus, 1758

##### ﻿ Acheta domesticus

Taxon classificationAnimaliaOrthopteraGryllidae

(Linnaeus, 1758)

08A632CC-A15F-588F-B1D0-1BD8AADEF57D

Figures 1 , 2 , 3 , 4 , 5 , 6 , 7 , 8 , 9 , 10 , 11 , Table 1

###### Material examined.

Pakistan, **Sindh Prov**. • 2♂, 8♀; Riffat, Surriya; 28 Aug. 2019; Mithi 24.7436°N, 69.8061°E, 11♂, 17♀; Riffat, Surriya; 30 Aug. 2019; Naushahro feroze 26.8463°N, 68.1253°E, 3♀; Surriya, Riffat; 3 Sep. 2019; Chachro 25.1156°N, 70.2557°E, 5♂, 11♀; Riffat, Surriya; 11 Sep. 2019; Umerkot 25.3549°N, 69.7376°E, 5♂, 16♀; Surriya, Riffat; 12 Sep. 2019; Nara 34.6851°N, 135.8048°E, 12♂, 24♀; Surriya, Riffat; 17 Sep. 2019; Nagarparkar 24.3572°N, 70.7555°E, 1♂, 4♀; 14 Aug. 2019; Tharparkar 24.8777°N, 70.2408°E, 2♂, 9♀; Riffat, Surriya; 16 Aug. 2019; Sanghar 26.0436°N, 68.9480°E, 1♂, 8♀; Riffat, Surriya; 17 Aug. 2019; Islamkot 24.7014°N, 70.1783°E.

###### Description.

**Figure 1.**
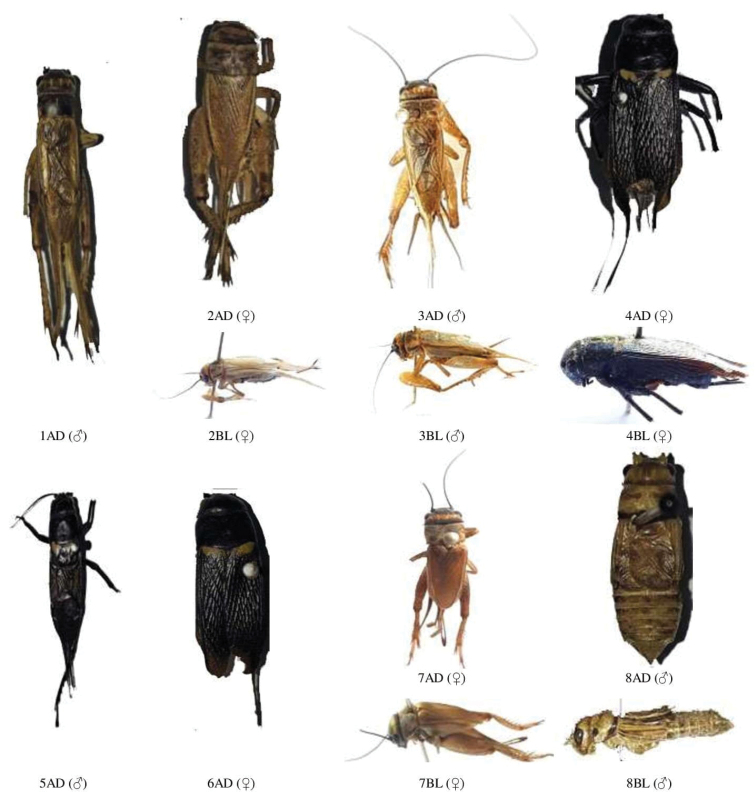
**A** male and female dorsal and lateral view of Gryllidae species. Subfamily Gryllinae: 1, 2 *Achetadomesticus* ♂♀, 3 *A.hispanicus* ♂, 4, 5 Gryllus (Gryllus) bimaculatus ♀♂, 6 G. (Gryllus) campestris ♀, 7 *G.septentrionalis* ♀, 8 *Gryllodessigillatus* ♂ **B** male and female dorsal and lateral view of Gryllidae species. Subfamily Gryllinae: 9 *Gryllodessupplicans* ♀, 10 Teleogryllus (Brachyteleogryllus) occipitalis ♀, 11,12 T. (Brachyteleogryllus) commodus ♂♀, 13 *Modicogryllussindhensis* sp. nov. ♀, 14 *Svercuspalmetorum* ♀ **C** male and female dorsal and lateral view of Gryllidae species. Subfamily Gryllinae: 15 *Miogryllusitaquiensis* ♀, 16 *Callogryllussaeedi* ♀, 17 *C.ovilongus* ♀, 18 *C.bilineatus* ♀, 19 *Lepidogryllussiamensis* ♀, Subfamily Oecanthinae: 20 *Oecanthusfultoni* ♀. Abbreviations: D, dorsal, L, lateral. Scale bars: 2 mm.

**Figure 2.**
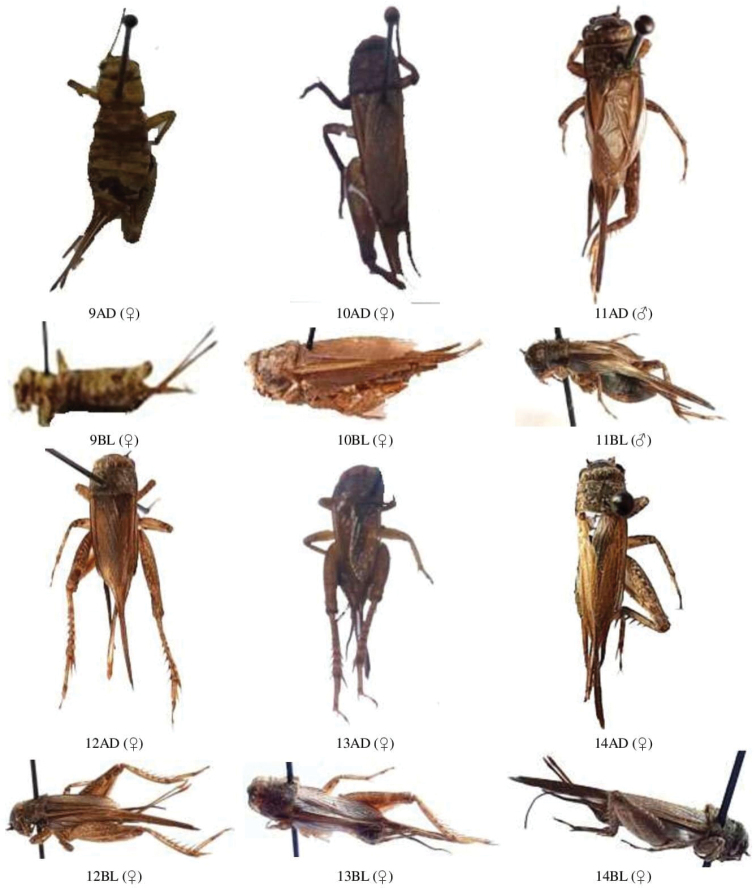
Male and female head dorsal view of Gryllidae species. Subfamily Gryllinae: 1, 2 *Achetadomesticus* ♂♀, 3 *A.hispanicus* ♂, 4, 5 Gryllus (Gryllus) bimaculatus ♂♀, 6 G. (Gryllus) campestris ♀, 7 *G.septentrionalis* ♀, 8-*Gryllodessigillatus* ♂, 9 *Gryllodessupplicans* ♀, 10 T. (Brachyteleogryllus) commodus ♂. Abbreviations: D, dorsal, L, lateral. Scale bars: 2 mm.

Medium size, pubescent and deep. General colouration light fulvous or testaceous (Fig. 1A). Head brown with two variables extending testaceous bands (Fig. 2A, B). Pronotum adorned with two large brown bands (Fig. 4A, B). Elytra extending to the apex of abdomen. Wings usually larger than the elytra (Fig. 8A, B). Legs yellowish with a few brown spots. Posterior tibia armed with eleven spines on the basal side (Fig. 6A, B. Ovipositor large and acute.

**Table 1.** Distribution of Gryllidae species in different areas of Sindh, with numbers collected at each locality.

Species	Mithi	Naushahro feroze	Chachro	Umerkot	Nara	Nagarkarkar	Tharparkar	Sanghar	Islamkot
*Achetadomesticus*	10	28	03	16	21	36	05	11	09
*Achetahispanicus*	01	—	—	—	—	—	—	—	—
Gryllus (Gryllus) bimaculatus	09	02	07	12	02	22	17	04	15
*G (Gryllus) campestris*	—	—	08	33	03	19	23	—	11
*Gryllusseptentrionalis*	—	—	—	01	—	—	—	—	—
Gryllodessigillatus	02	09	18	24	—	13	05	—	—
*Gryllodessupplicans*	—	—	—	01	02	—	—	—	—
*Callogryllussaeedi*	—	—	—	—	—	—	—	05	—
*Callogryllusovilongus*	—	—	—	—	—	04	—	—	—
*Callogryllusbilineatus*	—	—	—	—	—	—	—	—	02
*Modicogryllussindhensis*	—	—	—	01	—	—	—	—	—
Teleogryllus (Brachyteleogryllus) occipitalis	01	—	—	—	—	—	—	—	—
T.(Brachyteleogryllus) commodus	—	—	—	—	—	02	—	—	—
*Lepidogryllussiamensis*	—	—	—	01	—	—	—	—	—
*Svercuspalmetorum*	—	—	—	—	—	—	02	—	—
*Miogryllusitaquiensis*	—	—	01	—	—	—	—	—	—
*Oecanthusfultoni*	—	—	—	01	—	—	—	—	—

**Male**: LH 2.25 ± 0.15 (mm), LP 3.5 ±1.4 (mm), LT 4.5 ± 1.73 (mm), LF 11.0 ± 2.08 (mm), LT 6.01 ± 1.0 (mm), LT 4.9 (mm), TBL 15.33 ± 4.2 (mm) **Female**: LH 3.26 ± 2.8 (mm), LP 3.83 ± 1.50 (mm), LT 4.7 ± 1.23 (mm), LF 14.0 ± 4.11 (mm), LT 7.33 ± 2.06 (mm), LO 10.66 ± 2.94 (mm), TBL 16 ± 3.05 (mm).

###### Ecology.

*Achetadomesticus* is broadly distributed in the field. They complete their life cycle within 60–70 days. Agricultural crops affected by this species are *Tritiumaestivum* (wheat), *Oryzasativa* (rice), *Sacharumofficinarium* (sugarcane), and *Dactylocteniumaegyptium* (common lawn grasses).

###### Global distribution.

Czech Republic, Greece, Peloponnese, Patras, Yugoslavia, Serbia, USA, India, Pakistan (Cigliano et al. 2020).

###### Remarks.

*Achetadomesticus* is generally recognised as the house cricket, cosmopolitan in nature. The presence of this species was reported by Chopard (1969) from Himalayas, Srinagar, and Kashmir, at 6000 ft a.s.l. Previously, Ghouri (1961) stated that *A.domesticus* and other species were severe pests of many crops in Pakistan, and Malik (2012) also stated it from human habitation. At present we have recorded this species from Chachro (25.1156°N, 70.2557°E). We have collected large numbers of specimens from agricultural fields and confirm that it is a pest of various crops.

##### ﻿ Acheta hispanicus

Taxon classificationAnimaliaOrthopteraGryllidae

Rambur, 1838

4A2D2F62-3B00-57F2-819E-B96B66837114

Figures 1 , 2 , 3 , 4 , 5 , 6 , 7 , 8 , 9 , 10 , 11 , Table 1

###### Material examined.

Pakistan, **Sindh Prov.** • 1♂; Riffat, Surriya; 23 Aug. 2019; Mithi 24.7436°N, 69.8061°E.

###### Description.

Rather large and robust, colouration brownish-yellow (Fig. 1C). Head blackish with shining occiput (Fig. 2C). Pronotum unicolourous, concave, very slightly widening; anterior and posterior margins almost straight with numerous spots (Fig. 1C). Elytra extending to the apex of abdomen, mirror small, obliquely transverse (Fig. 8C). Wings long. Legs pale yellowish with numerous hairs. Tibia with eleven pointed spines on either side (Fig. 6C). Abdomen yellow, pubescent. Cerci well developed, pointed.

**Male**: LH 2.17 (mm), LP 2.66 (mm), LT 13 (mm), LF 11 (mm), LT 08 (mm), LT 4.9 (mm), TBL 28 (mm).

###### Ecology.

The species was recorded from Mithi. Usually, they are found in ditches of soil in rice fields. Weissman et al. (1980) reported that the adults seemed to appear in August but were abundant mid-August to September with a decline observed in October.

###### Global distribution.

Portugal, Spain: Granada, India, Pakistan (Cigliano et al. 2020).

###### Remarks.

This species is a new record from Sindh, Pakistan, and also for Asia. The body is wide and robust in structure compared to the more widely distributed *A.domesticus*. In our collection only a single male was captured, so more extensive collections are needed to establish its complete distribution.

#### Genus *Gryllus* Linnaeus (1758)

##### ﻿ Gryllus (Gryllus) bimaculatus

Taxon classificationAnimaliaOrthopteraGryllidae

De Geer, 1773

577137E1-D482-5BCB-8230-C2D70E0C80E8

Figures 1 , 2 , 3 , 4 , 5 , 6 , 7 , 8 , 9 , 10 , 11 , Table 1

###### Material examined.

Pakistan, **Sindh Prov.** • 5♂, 4♀; Surriya, Riffat; 21 Aug. 2019; Mithi 24.7436°N, 69.8061°E, 2♀; Riffat; Naushahro feroze 26.8463°N, 68.1253°E, 3♂, 4♀; Riffat, Surriya; 12 Sep. 2020; Chachro 25.1156°N, 70.2557°E, 4♂, 8♀; Surriya, Riffat; 19 Sep. 2020; Umerkot 25.3549°N, 69.7376°E, 2♀; Riffat; 20 Aug. 2020; Nara 34.6851°N, 135.8048°E, 6♂, 16♀; Surriya; 24 Aug. 2020; Nagarparkar 24.3572°N, 70.7555°E, 6♂, 11♀; Riffat, Surriya; 23 Aug. 2020; Tharparkar 24.8777°N, 70.2408°E, 1♂, 3♀; Riffat; 26 Aug. 2020; Sanghar 26.0436°N, 68.9480°E, 3♂, 8♀; Riffat, Surriya; 27 Aug. 2020; Islamkot 24.7014°N, 70.1783°E.

###### Description.

Large size, stout. Colour blackish. Head curved feebly at anterior; wider at posterior (Fig. 1D, E). Pronotum concave with piriform impression on anterior disc (Fig. 4D, E). Elytra reach to the top of abdomen, wings much long (Fig. 8D, E). Legs dark brown and strongly pubescent (Fig. 1D, E). Posterior femora rather thick, dark brown with rufous base; posterior tibia with eight spines on superior margin (Fig. 6D, E). Ovipositor rather long and slender, feebly curved with very narrow, smooth, acute apical valves (Fig. 1D, E).

**Male**: LH 2.25 ± 0.15 (mm), LP 3.45 ± 0.057 (mm), LT 4.1 ± 1.5 (mm), LF 14.5 ± 0.57 (mm), LT 11.0 ±1.15 (mm), LT 4.2 (mm), TBL 22.5 ± 0.57 (mm) **Female**: LH 4.76 ± 0.74 (mm), LP 4.66 ± 0.35 (mm), LT 4.5 ± 1.63 (mm), LF 15.33 ± 0.57 (mm), LT 11.66 ± 0.816 (mm), LO 18.5 ± 0.57 (mm), TBL 16 ± 3.05 (mm).

###### Ecology.

This species frequently occurred in the field. Plants affected by this species are *Tritiumaestivum* (wheat), *Oryzasativa* (rice), *Sacharumofficinarium* (sugarcane), and *Echinochloacolonum* (jungle rice). This species is hemimetabolous and moults 8–11 times to become adult (pers. obs.).

###### Global distribution.

Ukraine, France, Spain, USA, India, West Bengal, Kashmir, Pakistan, Mali (Cigliano et al. 2020).

###### Remarks.

*Gryllusbimaculatus* is variable in size with colour variations. During this study we collected this species from dry parts of Nagarparkar and confirm its presence in dry barren areas. Chopard (1969) reported that G. (Gryllus) bimaculatus causes severe damage to potato plants.

##### Gryllus (Gryllus) campestris

Taxon classificationAnimaliaOrthopteraGryllidae

Linnaeus, 1758

B6558B51-C1A6-5974-A4B8-509CB1E0C3F6

Figure 1 , 2 , 3 , 4 , 5 , 6 , 7 , 8 , 9 , 10 , 11 , Table 1

###### Material examined.

Pakistan, **Sindh Prov**. • 2♂, 6♀; Riffat; 12 Jul. 2019; Chachro 25.1156°N, 70.2557°E, 10♂, 23♀; Riffat, Surriya; 17 Jul. 2019; Umerkot 25.3549°N, 69.7376°E, 3♀; Riffat; 18 Aug. 2019; Nara 34.6851°N, 135.8048°E, 7♂, 12♀; Surriya, Riffat; 27 Aug. 2019; Nagarparkar 24.3572°N, 70.7555°E, 8♂, 15♀; Riffat, Surriya; 8 Jul. 2019; Tharparkar 24.8777°N, 70.2408°E, 4♂, 7♀; Surriya, Riffat; 3 Sep. 2020; Islamkot 24.7014°N, 70.1783°E.

###### Description.

A large species, rather similar to G. (Gryllus) bimaculatus, but more rounded and curved (Fig. 1F). Head yellowish brown with patches and raised veins (Fig. 2F). Pronotum convex above, blackish brown with fine greyish pubescent; posterior margin sinuated; elytra extending to the apex of the abdomen (Fig. 4F), legs blackish testaceous with brown spots, pubescent. Posterior femora rather short and thick; posterior tibia armed with six spines on each margin (unfortunately broken of during photography). Abdomen brown, ovipositor long, slender with narrow, very acute apical valves (Fig. 1F).

**Female**: LH 4.6 (mm), LP 4.9 (mm), LT 18 (mm), LF 15, LT 13, TBL 29 (mm).

###### Ecology.

*Tritiumaestivum* (wheat), *Oryzasativa* (rice), *Sacharumofficinarium* (sugarcane), *Echinochloacolona* (cultivated field) are all affected by this pest. It seems rare in numbers, and not widely occurring like other species of Gryllidae. These specimens were collected from rice fields whereas other plants such as sugarcane and wheat were also present, but with minor damage.

###### Global distribution.

Denmark, Germany, Netherlands, Switzerland, UK, Pakistan (Cigliano et al. 2020).

###### Remarks.

Due to its rare status and sporadic nature G. (G.) campestris is included in the red lists Hochkirch et al. (2007). It is flightless in its habitat of dune, short grasses, chalky soil, and light sandy porous soils. During our field survey we collected material from different districts. Our examination demonstrates that this species has morphological similarity to G. (Gryllus) bimaculatus but few differences in wing pattern and head morphology identifies each species.

##### ﻿ Gryllus septentrionalis

Taxon classificationAnimaliaOrthopteraGryllidae

F. Walker, 1869

1D491229-64AA-5FC3-9E00-14A6BBB567B4

Figures 1 , 2 , 3 , 4 , 5 , 6 , 7 , 8 , 9 , 10 , 11 , Table 1

###### Material examined.

Pakistan, **Sindh Prov.** • 1♀; Riffat, Surriya; 21 Jul. 2019; Mahendrani, Umerkot 25.3549°N, 69.7376°E.

###### Description.

Medium size, colouration rufous brown, rather strongly pubescent (Fig. 1G). Head long, rounded without any ornamentation. Face brown with yellow horizontal band; ocelli big, brown (Fig. 2G). Pronotum slightly enlarged in front, anterior margin feebly concave, posterior one pointed; disc convex, rufous with two large piriform impressions; lateral lobes with yellowish inferior part (Fig. 4G). Elytra brownish, reaching to apex of abdomen; dorsal fields with slightly oblique veins, rather projecting. Wings long (Fig. 9A). Legs pubescent; anterior and medium femora rufous brown; anterior tibia with large slender external tympanum; only internal face depressed. Posterior femora rather long, swollen. Tibia shorter than femora, armed with nine basal spines, four on joint of metatarsus (Fig. 6F). Abdomen brown; ovipositor moderately long, rather slender with very acute apical valves (Fig. 1G).

**Female**: LH 3.9 (mm), LP 4.2 (mm), LT 18 (mm), LF 12.5 (mm), LT 08 (mm), LT 05 (mm), TBL 26 (mm).

###### Ecology.

*Gryllusseptentrionalis* was collected from the village of Mahendrani, Umerkot in August.It was noted that this field was surrounded by *Citrus* (lemon) crops and other wild vegetation. This study suggests that extensive surveys are needed.

###### Global distribution.

Argentina, Paraguay, Caribbean, Jamaica, Pakistan (Cigliano et al. 2020).

###### Remarks.

This is the first record from the deserts of Thar, Sindh, Pakistan. According to Saeed (2000), this species of cricket occurs in terrestrial habitats throughout the world, and mostly damages cotton, rice, millet, and sugarcane crops. Due to their predatory nature, they are also helpful in biological control, but more detailed investigations are needed to identify this strategy in future.

#### Genus *Gryllodes* Saussure, 1874

##### ﻿Gryllodes sigillatus

Taxon classificationAnimaliaOrthopteraGryllidae

Walker, 1869

C09A11A4-FD65-5156-A641-4B21BB1554C9

Figures 1 , 2 , 3 , 4 , 5 , 6 , 7 , 8 , 9 , 10 , 11 , Table 1

###### Material examined.

Pakistan, **Sindh Prov.** • 2♀; Riffat; 14 Jul. 2020; Mithi 24.7436°N, 69.8061°E, 1♂, 8♀; Surriya, Riffat; 19 Jul. 2020; Naushahro feroze 26.8463°N, 68.1253°E, 3♂, 15♀; Riffat; 2 Sep. 2019; Chachro 25.1156°N, 70.2557°E, 9♂, 12♀; Riffat, Surriya; 13 Aug. 2020; Umerkot 25.3549°N, 69.7376°E, 6♂, 7♀; Surriya, Riffat; 16 Aug. 2020; Nagarparkar 24.3572°N, 70.7555°E, 5♀; Riffat, Surriya; 4 Sep. 2020; Tharparkar 24.8777°N, 70.2408°E.

###### Description.

Medium size, depressed, rather strongly pubescent (Fig. 1H). Head brown with wider, transverse yellowish bands on dorsal field; anterior narrow band curved between ocelli; face short, yellow; clypeus spotted with brown, front with feeble suture (Fig. 2H). Pronotum transverse with concave anterior margin; disc almost straight; yellowish with wide brown band along posterior margin and a more or less important spot of the same colour on the impressus (Fig. 4H). Elytra extending to 1/3 of abdominal tergite, truncated, rounded at apex; mirror quite apical, little wider than long, rounded posteriorly; wings reduced (Fig. 9B). Abdomen brown in the male (Fig. 1H).

**Male**: LH 2.8 ± 0.72 (mm), LP 3.25 ± 0.62 (mm), LT 4.1 ± 5.2 (mm), LF 11.5 ±1.0 (mm), LT 8.0 ± 0.57 (mm), TBL 14.5 ± 1.0 (mm) **Female**: LH 2.10 ± 0.8 (mm), LP 3.32 ± 0.72 (mm), LT 4.3 ± 5.7 (mm), LF 12.5 ± 1.2 (mm), LT 8.2 ± 0.62 (mm), TBL 18.6 ± 2.1 (mm).

###### Ecology.

It commonly found everywhere but surprisingly, a single male only was reported during the present survey. Usually, this species is found in homes and lives under bricks and debris, and also in kitchens.

###### Global distribution.

Australasia, Australia, Malaysia, West Bengal, USA, India, Pakistan (Cigliano et al. 2020).

###### Remarks.

*Gryllodessigillatus* is cosmopolitan in nature. This species is generally known as the tropical house cricket or Indian house cricket because they are found everywhere, domestic in all tropical countries. Khan (1954) reported that it caused huge damage to textiles mills in India. During our field survey we observed that this species moves at dusk from the holes of a termite mound. However, this species is not termitophilous in nature like other insects; it does not live with the termites.

##### ﻿ Gryllodes supplicans

Taxon classificationAnimaliaOrthopteraGryllidae

(Walker, 1859)

7D366D6B-30AE-57D2-82DA-8BDD851EDAD4

Figures 1 , 2 , 3 , 4 , 5 , 6 , 7 , 8 , 9 , 10 , 11 , Table 1

###### Material examined.

Pakistan, **Sindh Prov.** • 2♀; Riffat; 3 Jul. 2019; Nara 34.6851°N, 135.8048°E, 1♀; Surriya; 4 Jul. 2019; Umerkot 25.3549°N, 69.7376°E.

###### Description.

Medium size, yellowish brown (Fig. 1I). Head small, narrow at the anterior, slightly curved at posterior. Face short, yellow with spotted clypeus. Frontal suture feebly arched (Fig. 2I). Pronotum transverse, feebly concave at anterior (Fig. 4I). Female elytra equilateral, reduced, extending to the extremity of abdomen. Wings caudate (Fig. 9C). Legs pubescent, yellowish, with few brown spots. Anterior tibia perforated on the external face with a rather long, oval tympanum (Fig. 6H). Abdomen brown with triangular median line on dorsal field. Ovipositor long, straight with narrow lanceolate apical valves (Fig. 1I).

**Female**: LH 3.15 (mm), LP 3.15 (mm), LT 4.2 (mm), LF 14 (mm), LT 10 (mm), LO 15 (mm), TBL 20 (mm).

###### Ecology.

Annandale (1924) reported that this species lives in crevices, mostly occurring in wood and frequently in holes of bungalows. During the present study, we collected this species from a stack of wood from Umerkot.

Khan (1954) noticed that all females of Gryllidae deposit more than 150 eggs when temperatures are favourable, between 20–25 °C with the relative humidity of 80–82%. At present, only females were captured and is longer in total body length (20 mm) with the ovipositor ca. 15 mm compared to Chopards’ (1969) report of total body length 12–15 mm and ovipositor 12–12.5 mm. This may be a geographical variant of the region; however, a detailed and comprehensive analysis of the taxa will be undertaken when more material will be collected.

###### Global distribution.

America, Singapore, Berlin, Ceylon, India, Malaysia, China, Sri-Lanka, and Pakistan (Cigliano et al. 2020).

###### Remarks.

Earlier, this species was collected by Chopard (1969) from various localities of India, but his specimens were smaller in size. The elytra of this species are longer than those of *Sigillatus*, leading to the question of whether this species could be a macropterous form of the proceeding one. Considering the extreme reduction of the elytra of the female of *Sigillatus*, it seems difficult to admit the possibility of a return to fully winged form. However, future studies with more samples should resolve this problem.

#### Genus *Teleogryllus* Chopard, 1961

##### ﻿ Teleogryllus (Brachyteleogryllus) occipitalis

Taxon classificationAnimaliaOrthopteraGryllidae

(Serville, 1838)

AE991E31-383D-5ED6-AF5B-D13C1662BB5F

Figures 1 , 2 , 3 , 4 , 5 , 6 , 7 , 8 , 9 , 10 , 11 , Table 1

###### Material examined.

Pakistan, **Sindh Prov.** • 1♀; Riffat; 5 Sep. 2019; Mithi 24.7436°N, 69.8061°E.

###### Description.

Medium to large size. Body pale brown (Fig. 1J). Head brown to dark with horizontal band at posterior margin. Ocelli dark brown (broken off while capturing photos). Pronotum dark brown, enlarged in front, its surface is rather strongly punctuated with numerous testaceous rufous spots (Fig. 4J). Female elytra extending to the apex of abdomen; elytral veins oblique, regularly spaced. Wings well developed with geometrical designs (Fig. 9D). Legs of the same colour as body; posterior femora moderately swollen, striated on external face; posterior tibiae armed with seven spines on each margin (Fig. 6I). Abdomen pale brown, yellowish beneath. Ovipositor long, slender (Fig. 1J).

**Female**: LH 2.1 (mm), LP 3.85 (mm), LT 08 (mm), LF 9 (mm), TBL 20 (mm).

###### Ecology.

*Teleogryllus* is commonly known as black field cricket. Species of this genus are reported as a serious pasture pest in Australia and the warmer northern regions of New Zealand (Banfield and Cottier 1948; Reynolds and Langton 1973; Mill 1978). They reported that each year black field crickets cause considerable losses in pasture production over the dry summer period when stock feed is short. The resulting bare areas in the pasture are then opened to weed invasion because the black field crickets consume only pasture seed.

During the present study we captured only a single female from *Loliumperenne* grasses, which is considered as perennial ryegrass pasture, the main feed for dairy cows in temperate regions. This study suggests that preference of crickets for perennial ryegrass may lead high risk of damage to cultivated areas of Pakistan.

###### Global distribution.

Sumatra, Java, Borneo, Philippines, Vietnam, Australia, Celebes, India, Bangladesh, Sri Lanka, Nepal, China, Burma, Malaysia, Singapore, Thailand, Pakistan (Cigliano et al. 2020).

###### Remarks.

Until now 52 species of *Teleogryllus* were recorded by Cigliano et al. (2020). Gorochov (1985) reviewed the *Teleogryllus* species from Asia and established two subgenera. He moved *T.occipitalis* (Serville, 1838), *T.emma* (Ohmachi & Matsuura, 1951*T.infernalis* (Saussure, 1877), *T.commodus* (Walker, 1869), and *T.oceanicus* (Le Guillou, 1841) into the subgenus Brachyteleogryllus with *T.occipitalis* as the type species, and he moved *T.mitratus* and *T.derelictus* into the subgenus Macroteleogryllus with the first as type species. Gorochov (1988) established another subgenus, *Afroteleogryllus*, with *T.clarus* as its type species from Africa, and added a further two new species in 1990. Otte (2006) downgraded genus *Cryncoides* as a subgenus under *Teleogryllus*. The remaining species are still in the pool of the subgenus Teleogryllus without having been studied again. In China, these crickets are often confused, and different species names have been used, until Ma et al. (2015) distinguished them by their genitalia. However, these changes are mainly based on morphological studies without molecular evidence.

##### ﻿ Teleogryllus (Brachyteleogryllus) commodus

Taxon classificationAnimaliaOrthopteraGryllidae

(Walker, 1869)

61F8D7AC-C21F-52AF-9A5F-DA9A92B7DAA9

Figures 1 , 2 , 3 , 4 , 5 , 6 , 7 , 8 , 9 , 10 , 11 , Table 1

###### Material examined.

Pakistan, **Sindh Prov.** • 1♂, 1♀; Riffat, Surriya; 19 Aug. 2019; Nagarparkar 24.3572°N, 70.7555°E.

###### Description.

**Figure 3.**
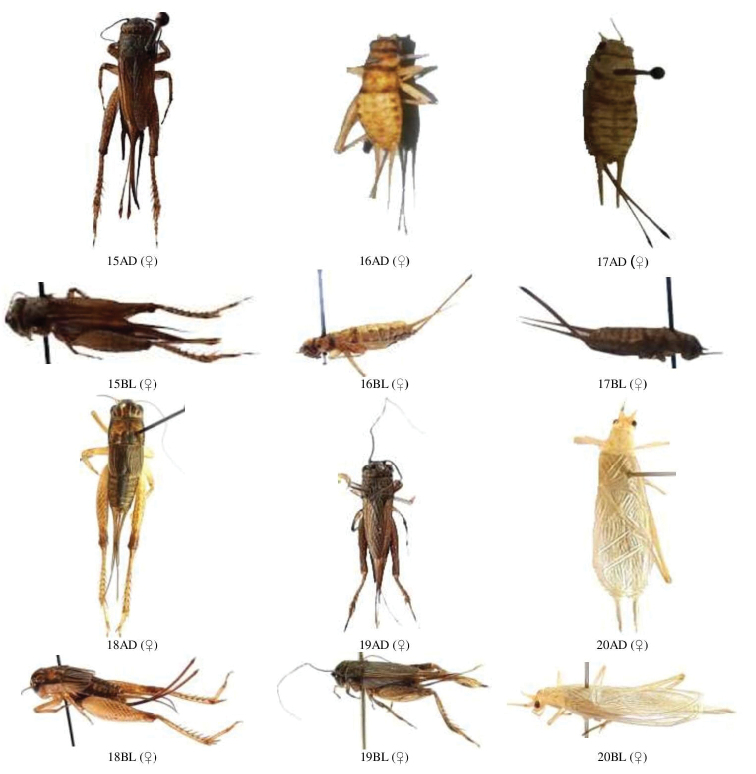
Male and female head dorsal view of Gryllidae species, subfamily Gryllinae: 11 T. (Brachyteleogryllus) commodus ♀, 12 *Modicogryllussindhensis* sp. nov. ♀,13 *Svercuspalmetorum* ♀, 14 *Miogryllusitaquiensis* ♀, 15 *Callogryllussaeedi* ♀, 16 *C.ovilongus* ♀, 17 *C.bilineatus* ♀, 18 *Lepidogryllussiamensis* ♀. Subfamily Oecanthinae: 19 *Oecanthusfultoni* ♀. Abbreviations: D, dorsal, L, lateral. Scale bars: 2 mm.

Head short with vertical pale and dark bands at posterior margin (Fig. 1K, L). Ocelli dorsal field with dark horizontal band (Figs 2J, 3A). Pronotum dark brown, more or less varied fulvous, with black inferior margin (Fig. 5A, B). Elytra extending to the second last segment of abdominal tergite, a little rounded at apex; dorsal field shiny brown with a narrow yellowish band along external and apical margins; mirror reduced and somewhat broad. Wing long, extending to apex of abdomen (Fig. 9E, F). Legs rather short, widened, yellowish, mottled with brown and covered with abundant brown pubescence in which are mixed long bristles. Tibia rather thin, longer than femora, armed with seven internal spines (Fig. 6J, K). Abdomen pale brown with dark coloured. Ovipositor long, straight, with feebly flattened, acute apical valves, (Fig. 1K, L).

**Figure 4.**
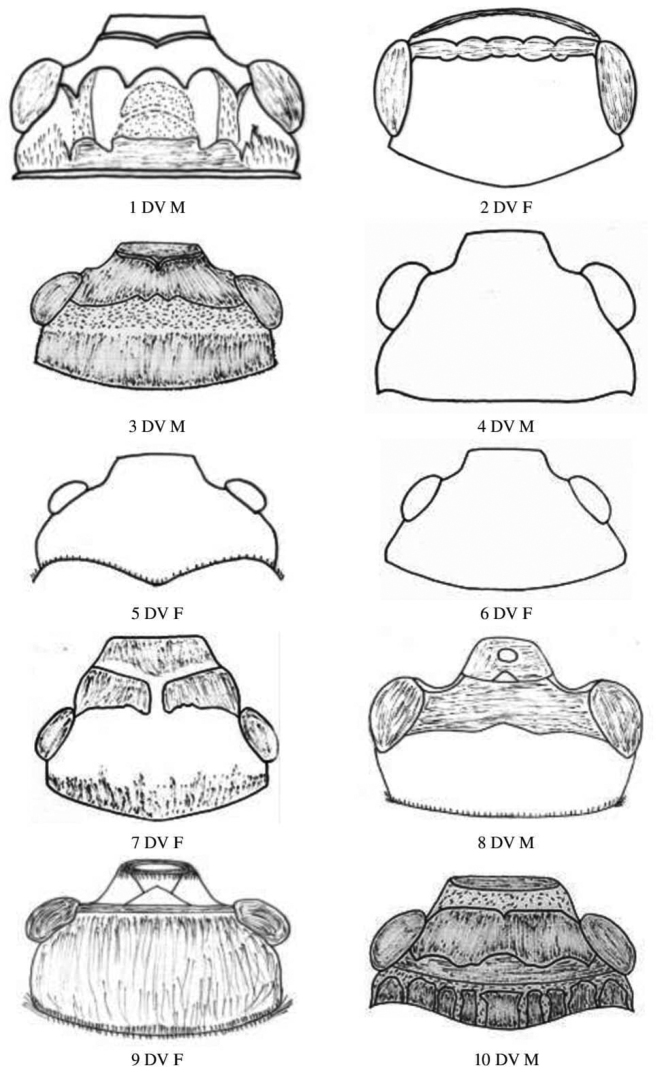
Male and female pronotum dorsal view of Gryllidae species, subfamily Gryllinae: 1, 2 *Achetadomesticus* ♂♀, 3 *A.hispanicus* ♂, 4, 5 Gryllus (Gryllus) bimaculatus ♂♀, 6 G. (Gryllus) campestris ♀, 7 *G.septentrionalis* ♀, 8 *Gryllodessigillatus* ♂, 9 *Gryllodessupplicans* ♀, 10 Teleogryllus (Brachyteleogryllus) occipitalis ♀. Abbreviations: D, dorsal, L, lateral. Scale bars: 2 mm.

**Male**: LH 4.34 (mm), LP 4.06 (mm), LT 14 (mm), LF 12.6 (mm), LT 7.7 (mm), LT 07 (mm), TBL 21 (mm), **Female**: LH 2.5 (mm), LP 3.1 (mm), LT 11 (mm), LF 08 (mm), LT 7.4 (mm), LT 04 (mm), TBL 17 (mm).

###### Ecology.

This species was reported from Nagarparkar. This area is surrounded by rock and fine sand. It was observed that due to burrowing habits this species uprooted many valued plants. This species is here reported from *Cymbopogoncommutatus* which are perennial grasses and mostly used for medicinal purposes in the locality.

###### Global distribution.

Australia, New Zealand, India, Pakistan (Cigliano et al. 2020).

###### Remarks.

This species is commonly known as black field cricket. Its powerful legs are used for jumping. This species has numerous white strips on the abdomen which make it different from the other species. Zalitschek et al. (2012) reported that they are omnivores in nature. However, dietary requirements are similar but perform different functions depending upon the sex of the specimen: females take a protein-rich diet for the production of eggs while, male requires it for producing mating calls to attract females.

#### Genus *Modicogryllus* Chopard, 1961

##### ﻿ Modicogryllus sindhensis sp. nov.

Taxon classificationAnimaliaOrthopteraGryllidae

68C31FBC-BEE6-5FAF-A5DB-7B96F715D918

http://zoobank.org/E85E40CA-489A-41AA-9C18-94A8D0677CFC

Figures 1 , 2 , 3 , 4 , 5 , 6 , 7 , 8 , 9 , 10 , 11 , Table 1

###### Material examined.

***Holotype*.** Pakistan, Sindh Prov. • 1♀; Riffat, Mohan leg.; 23 July 2019; Umerkot 25.3549°N, 69.7376°E. Reg. no.: 723 SEMJ.

###### Diagnosis.

This species has a brightly coloured body along with a shiny pronotum. The tegmina and wing show different patches on their entire surface.

###### Description.

**Figure 5.**
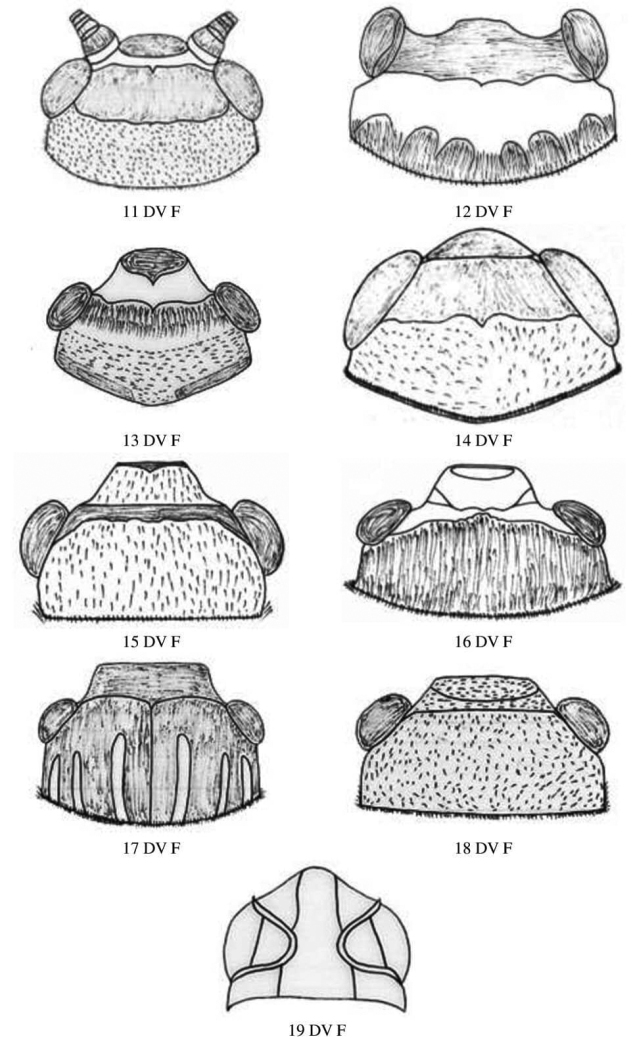
Male and female pronotum dorsal view of Gryllidae species, subfamily Gryllinae: 11, 12 T. (Brachyteleogryllus) commodus ♂♀, 13 *Modicogryllussindhensis* sp. nov. ♀,14 *Svercuspalmetorum* ♀, 15 *Miogryllusitaquiensis* ♀, 16 *Callogryllussaeedi* ♀, 17 *C.ovilongus* ♀,18 *C.bilineatus* ♀, 19 *Lepidogryllussiamensis* ♀, Subfamily Oecanthinae: 20 *Oecanthusfultoni* ♀. Abbreviations: D, dorsal, L, lateral. Scale bars: 2 mm.

**Figure 6.**
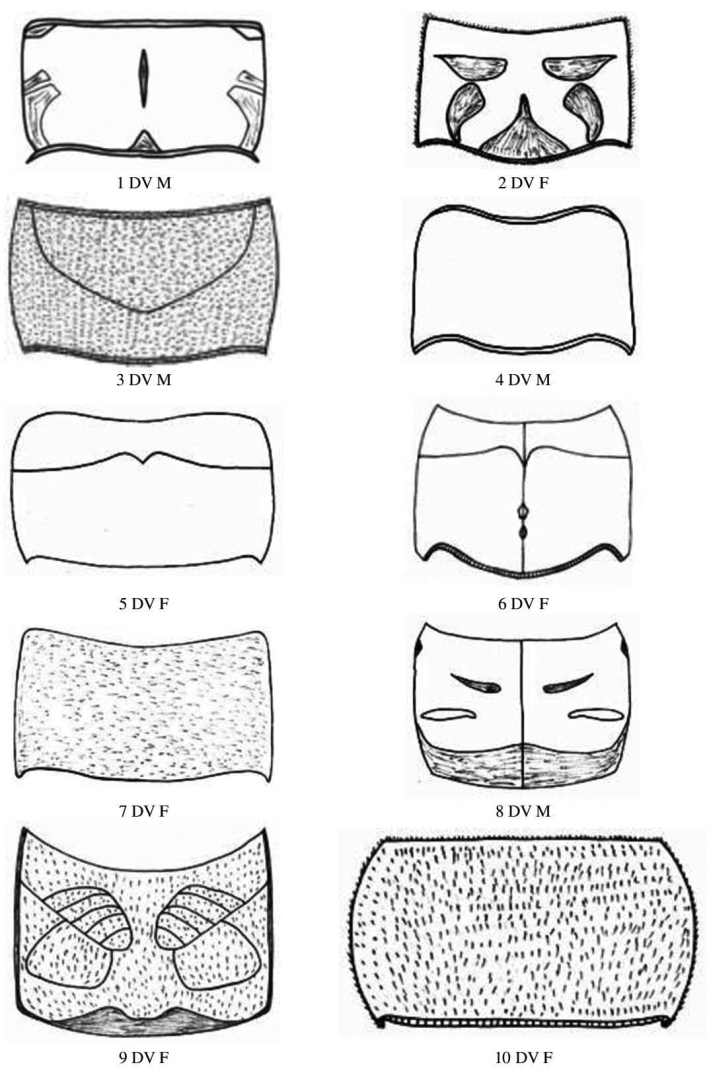
Femur and Tibia dorsal view of Gryllidae species, subfamily Gryllinae: 1, 2 *Achetadomesticus* ♂♀, 3 *A.hispanicus* ♂, 4, 5 Gryllus (Gryllus) bimaculatus ♂♀, 6 *G.septentrionalis* ♀, 7 *Gryllodessigillatus* ♂, 8 *Gryllodessupplicans* ♀, 9 Teleogryllus (Brachyteleogryllus) occipitalis ♀, 10, 11 T. (Brachyteleogryllus) commodus ♂♀, 12 *Modicogryllussindhensis* sp. nov. ♀. Abbreviations: D, dorsal, L, lateral. Scale bars: 2 mm.

Small size, covered in pubescence. Colour light brown (Fig. 1M). Head short, yellow, adorned with rufous spots, dorsal field of ocelli with pubescent horizontal dark bands (Fig. 3B). Pronotum depressed above with straight yellowish posterior margin on dorsal field (Fig. 5C). Elytra extending to apex of abdomen; veins of dorsal field rather irregular and condensed (Fig. 9G). Legs brownish. Pubescence rather thick, compressed. Anterior tibia bearing small, oval, external tympanum. Posterior tibia armed with ten external and one medio-internal spines (Fig. 6L). Abdomen brown. Ovipositor short, straight, slender with very small, lanceolate, acute apical valves (Fig. 1M).

**Female**: LH 2.1 (mm), LP 2.45 (mm), LF 10 (mm), LT 11(mm), LO 10 (mm), TBL 15 (mm).

###### Habitat.

The specimen was collected from *Sorghumvulgare* near Desert Thar (Umerkot) 25.3549°N, 69.7376°E.

###### Derivatio nominis.

The specific epithet refers to collection of this species from Desert Thar of Sindh.

###### Depository.

The type material (TN: 723 SEMJ) has been deposited in Sindh Entomological Museum, Department of Zoology, University of Sindh, Jamshoro.

###### Remarks.

The genus *Modicogryllus* was erected by Chopard (1961), within which he described four species from north-east part of India viz: *M.semiobscurus* (Chopard), *M.ehsani* (Chopard), *M.rehni* (Chopard), and *M.minimus* (Chopard). Our collected species has a brightly coloured body along with a shiny pronotum. The tegmina and wing show different patches on their entire surface. However, the shape, length, and other characteristics of the ovipositor make it different from the other species in the genus.

#### Genus *Svercus* Gorochov, 1988

##### ﻿ Svercus palmetorum

Taxon classificationAnimaliaOrthopteraGryllidae

(Krauss, 1902)

6E36839B-89EC-5A33-951E-C6C22C47F124

Figures 1 , 2 , 3 , 4 , 5 , 6 , 7 , 8 , 9 , 10 , 11 , Table 1

###### Material examined.

Pakistan, **Sindh Prov.** • 2♀; Surriya, Riffat; 22 Aug. 2020; Dahli, Tharparkar 24.8777°N, 70.2408°E.

###### Description.

**Figure 7.**
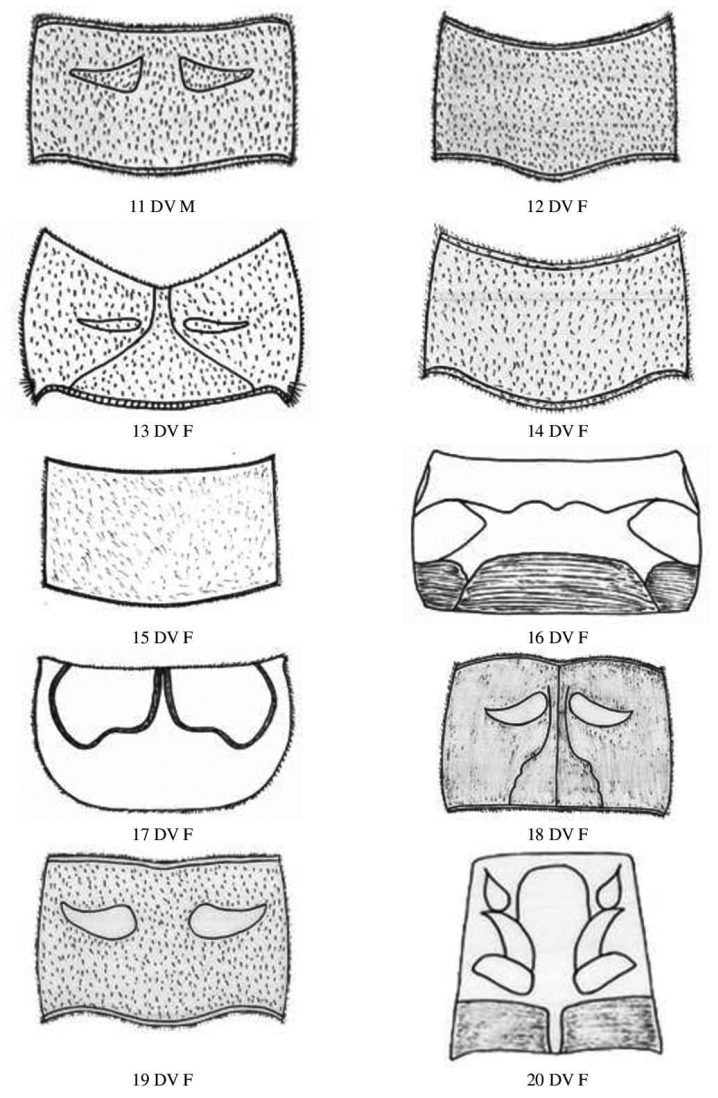
Femur and tibia dorsal view of Gryllidae species, subfamily Gryllinae: 13 *Svercuspalmetorum* ♀, 14 *Miogryllusitaquiensis* ♀, 15 *Callogryllussaeedi* ♀, 16 *C.bilineatus* ♀, 17 *Lepidogryllussiamensis* ♀, Subfamily Oecanthinae: 18 *Oecanthusfultoni* ♀. Abbreviations: D, dorsal, L, lateral. Scale bars: 2 mm.

Medium size. Colouration rufous brown, shiny (Fig. 1N). Head little wider than pronotum in front; occiput convex with frontal rostrum narrow, ocelli united by a small oblique keel (Fig. 3C). Pronotum dark brown, slightly broader than long with concave anterior margin, posterior margin feebly convex (Fig. 5D). Elytra extending to the apex of abdomen, narrow posteriorly. Wing well developed (Fig. 10A). Legs testaceous brown, pubescent. Anterior tibia perforated on external face only. Posterior tibia armed with nine internal, 11 external, one medio-internal spines (Fig. 7A). Abdomen brown. Ovipositor rather long, straight with lanceolate apical valves (Fig. 1N).

**Female**: LH 1.8 (mm), LP 2.7 (mm), LT 9.6 (mm), LF 09 (mm), LT 6.6 (mm), LT 03 (mm), TBL 16 (mm).

###### Ecology.

This species was collected from the village Dahli Taluka Tharparkar Sindh, Pakistan. This species was reported from *Larreatridentate* called the creosote bush. It is a medium-sized evergreen shrub with pointed leaves and a waxy coating. This plant has great medicinal value, recommended to cure fever, colds, stomach, pains, arthritis, and as a general pain killer; it is also used for cuts, and bacterial and fungal infections.

###### Global distribution.

Libya, Algeria, Pakistan (Cigliano et al. 2020).

###### Remarks.

Reitmeier et al. (2012) reported this species from Corsica in humid places (except those that were recorded from Bonifacio and Filitosa in September 2010. They further identified the status of this species, its distribution, and life parameters. During our field survey we also noticed that this species occurs in humid places, but we were not able to study its life parameters.

**Figure 8.**
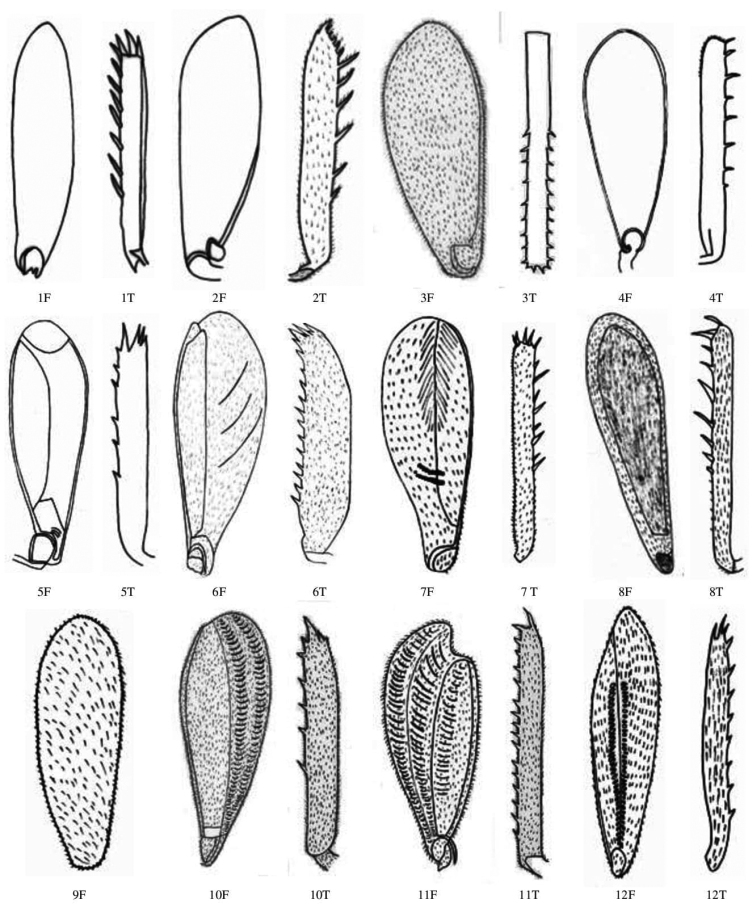
Male and female tegmen dorsal view of Gryllidae species, subfamily Gryllinae: 1, 2 *Achetadomesticus* ♂♀, 3 *A.hispanicus* ♂, 4, 5 Gryllus (Gryllus) bimaculatus ♂♀, 6 G. (Gryllus) campestris ♀. Abbreviations: D, dorsal, L, lateral. Scale bars: 2 mm.

**Figure 9.**
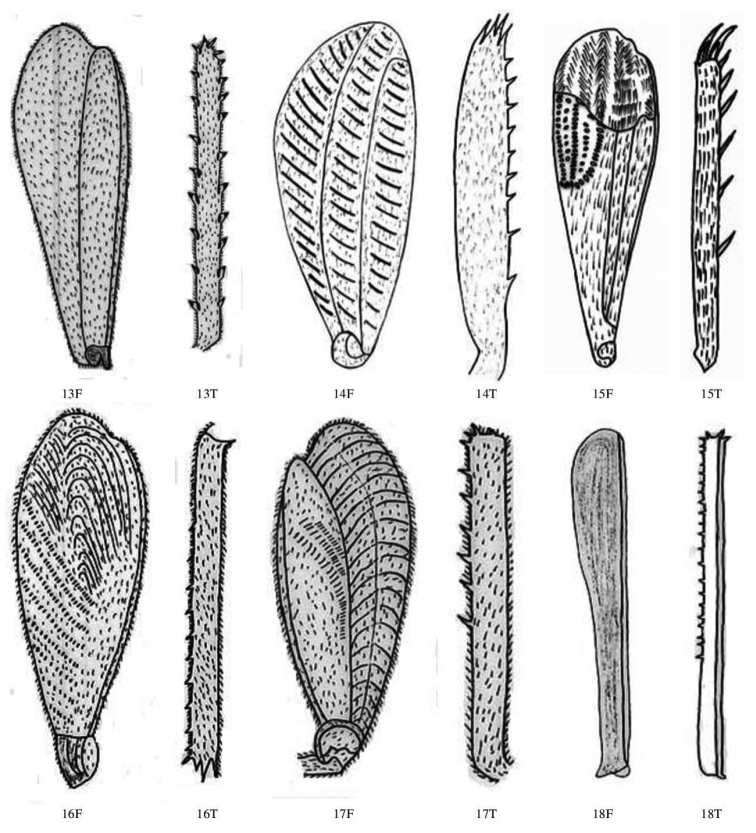
Male and female tegmen dorsal view of Gryllidae species, subfamily Gryllinae: 7 *G.septentrionalis* ♀, 8 *Gryllodessigillatus* ♂, 9 *GryllodesSupplicans* ♀, 10 Teleogryllus (Brachyteleogryllus) occipitalis ♀, 11, 12 T. (Brachyteleogryllus) commodus ♂♀, 13 *Modicogryllussindhensis* sp. nov. ♀. Abbreviations: D, dorsal, L, lateral. Scale bars: 2 mm.

**Figure 10.**
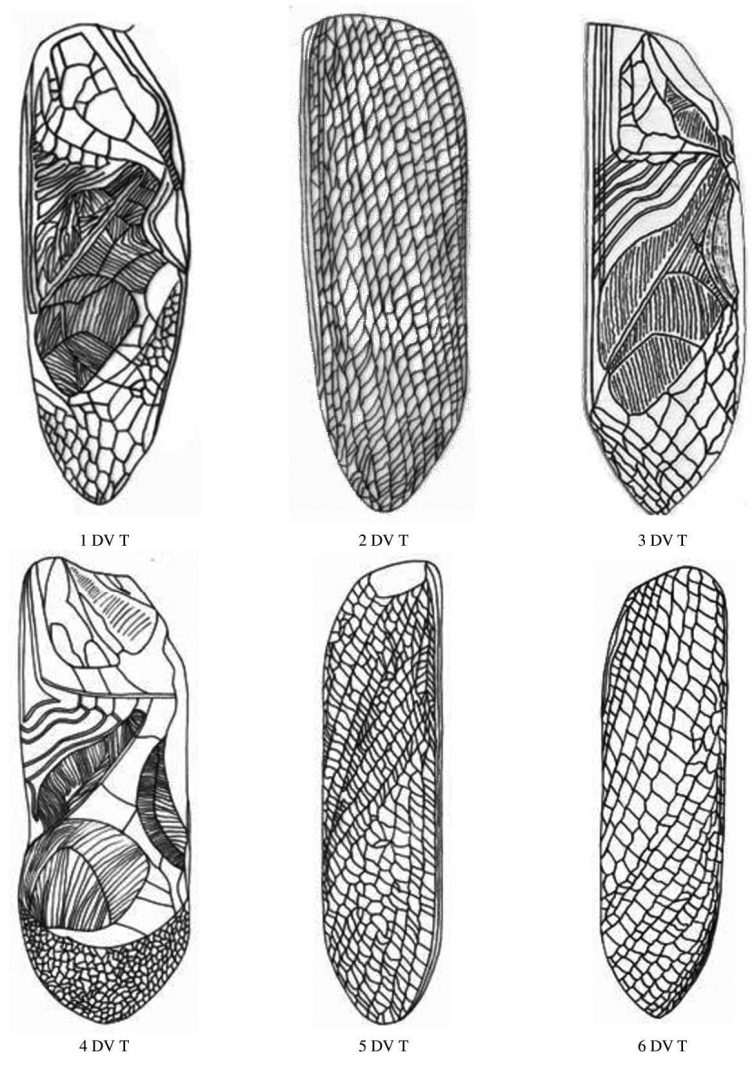
Male and female tegmen dorsal view of Gryllidae species, subfamily Gryllinae: 14 *Svercuspalmetorum* ♀, 15 *Miogryllusitaquiensis* ♀, 16 *Callogryllussaeedi* ♀, 17 *C.ovilongus* ♀,18 *C.bilineatus* ♀, 19 *Lepidogryllussiamensis* ♀, Subfamily Oecanthinae: 20 *Oecanthusfultoni* ♀. Abbreviations: D, dorsal, L, lateral. Scale bars: 2 mm.

**Figure 11.**
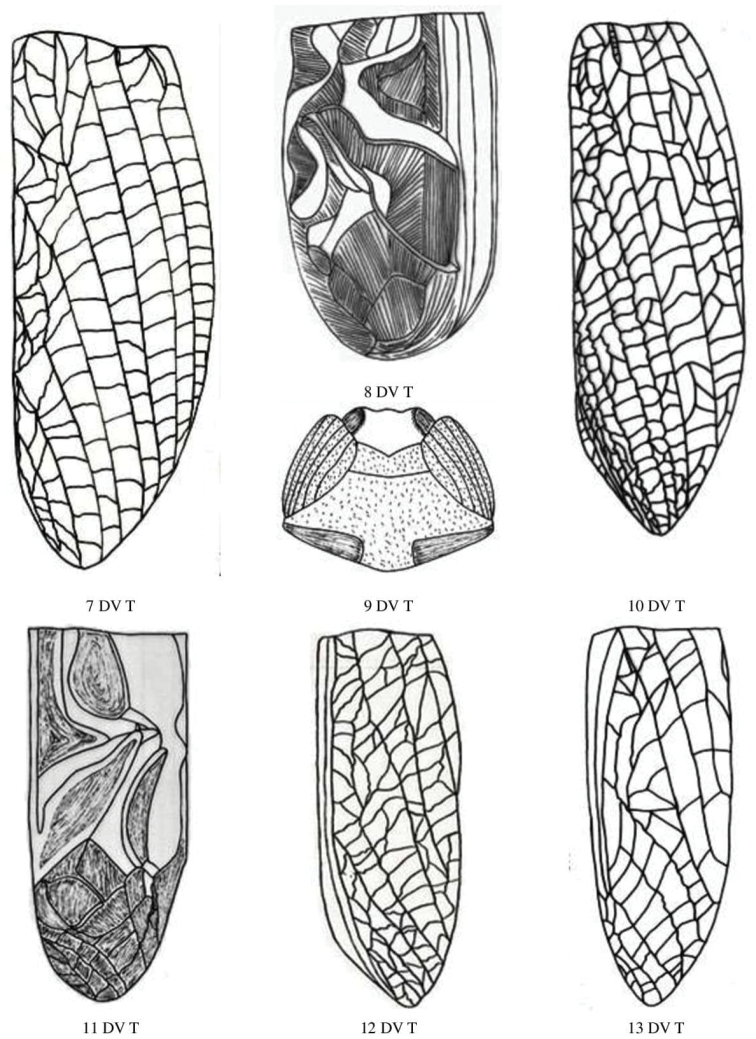
**A** map of Pakistan **B** map of Sindh province **C** areas within Sindh province. Maps reproduced by ArcGIS 10.5.

#### Genus *Miogryllus* Saussure, 1877

##### ﻿ Miogryllus itaquiensis

Taxon classificationAnimaliaOrthopteraGryllidae

Orsini & Zefa, 2017

0D4A560B-281E-5E78-807B-06939200545F

Figures 1 , 2 , 3 , 4 , 5 , 6 , 7 , 8 , 9 , 10 , 11 , Table 1

###### Material examined.

Pakistan, **Sindh Prov.** • 1♀; Riffat; 5 Sep. 2019; Chachro, Nagarparkar 24.3572°N, 70.7555°E.

###### Description.

Medium size. Colouration brown (Fig. 1O). Head black bright and globous; whitish spot posteriorly containing scape and following inner margins of eyes, becoming punctuated with brown with white stripe before reaching occiput (Fig. 3D). Pronotum black with pubescence, dorsal disc wider than long, bristles on anterior and posterior margins; lateral lobes marked with antero-ventral whitish spot which becomes pale brown posteriorly (Fig. 5E). Elytra extending to two-thirds of abdomen, apical field well developed. Wing surpassing abdomen tip (Fig. 10B). Legs dark brown dorsally, whitish ventrally. Tibia armed with nine internal, four medio-internal spines (Fig. 7B). Abdomen black, sternites whitish. Cerci pale brown, short. Ovipositor long, slender, straight with lanceolate apical valves (Fig. 1O).

**Female**: LH 03 (mm), LP 3.1 (mm), LT 09 (mm), LF 10 (mm), LT 0.8 (mm), LT 4.2 (mm), TBL 12 (mm).

###### Ecology.

This species was reported from Chachro, Nagarparkar on *Enceliafarinose* roots. This plant is commonly known as the Brittle bush. It is a medium-sized, rounded shrub with long, oval, silvery grey leaves. The resin collected from this plant is used as glue (Hogan and Michael 2013); these authors also stated that Brittle bush treats toothaches. Some animals such as desert Bighorn sheep and Kangaroo rats eat its seeds.

###### Global distribution.

Argentina, Brazil South, Rio Grande do Sul, Itaqui, Sindh, Pakistan (Cigliano et al. 2020).

###### Remarks.

The pronotum of *M.itaquiensis* bears a whitish lateral lobe, while *M.tucumanensis* has the pronotum with uniform colouration. We collected a single female for the first time from Chachro, Sindh, Pakistan. However, more extensive surveys are needed to explore its distribution in the desert region.

#### Genus *Callogryllus* Sjöstedt, 1910

##### ﻿ Callogryllus saeedi

Taxon classificationAnimaliaOrthopteraGryllidae

(Saeed, 2000)

78D3AB0D-B679-5D37-BAA7-41B878242556

Figures 1 , 2 , 3 , 4 , 5 , 6 , 7 , 8 , 9 , 10 , 11 , Table 1

###### Material examined.

Pakistan, **Sindh Prov.** • 5♀; Surriya, Riffat; 23 Aug. 2020; Sanghar 26.0436°N, 68.9480°E.

###### Description.

Medium size. Colouration yellow (Fig. 1P). Head short, narrow, yellowish shiny, adorned on each side with dark brown line extending from occiput, along eye (Fig. 3E). Pronotum as wide as long, barely widening anteriorly with two dark spots on dorsal field (Fig. 5F). Elytra reduced. No wings (Fig. 10C). Legs yellowish, strongly pubescent. Anterior tibia perforated with oval tympanum on external face. Posterior femora rather thick, brown with rufous base, posterior tibia armed with six long external, four various medio-internal spines (Fig. 7C). Abdomen yellow with dark spots on each tergite. Ovipositor long, straight, slender (Fig. 1P).

**Female**: LH 2.1 (mm), LP 2.8 (mm), LT 03 (mm), LF 12 (mm), LT 10 (mm), LO 14 (mm), TBL 17 (mm).

###### Ecology.

This species was previously reported by Saeed (2000) from *Triticumaestivum* in Pakistan. We reported the female from *Dactylocteniumaegyptium* grasses.

###### Global distribution.

India (this study), Pakistan (Saeed et al. 2000).

###### Remarks.

During this study, we have reported five females from Sanghar District which are a new record for Sindh province. Our thorough examination shows that this species is similar to *C.ovilongus* with the exception of a dark slanting band between the compound eyes, and the size of ovipositor: *C.saeedi* has a smaller ovipositor which is ca. 14 mm while *C.ovilongus* has a longer ovipositor, ca. 18–20 mm. In addition, the elytra of this female are quite different from those of *C.ovilongus*.

##### ﻿ Callogryllus ovilongus

Taxon classificationAnimaliaOrthopteraGryllidae

Saeed & Yousuf, 2000

383C6267-8142-5FB4-B169-758569BF4226

Figures 1 , 2 , 3 , 4 , 5 , 6 , 7 , 8 , 9 , 10 , 11 , Table 1

###### Material examined.

Pakistan, **Sindh Prov.** • 4♀; Riffat, Surriya; 16 Sep. 2020; Nagarparkar 24.3572°N, 70.7555°E.

###### Description.

Medium size. Colouration yellow (Fig. 1Q). Head short, narrow, very neat. Eyes rounded, moderately projecting; ocelli small (Fig. 3F). Pronotum 1.5 × as wide as long, slightly concave at anterior margin, straight at posterior margin; one side rather strongly convex (Fig. 5G). Elytra yellow, reduced (Fig. 10D). No wings. Legs light yellow, hind femora thick at base and slightly narrow at posterior, armed with six internal spines. Hind tibiae small, narrow, and straight. Abdomen dark yellowish above, pubescent and pale yellow beneath. Ovipositor rather long, very slender with extremely narrow, acute apical valves (Fig. 1Q).

**Female**: LH 3.85 (mm), LP 3.5 (mm), LT 5.2 (mm), LF 4.1 (mm), LO 15 (mm), TBL 16 (mm).

###### Ecology.

During the present study, females of this species are reported from Nagarparkar, Desert Thar, from xerophytic plants which were surrounded by sagebrush and saltbush trees.

###### Global distribution.

China, India, Bangladesh, Nepal, Pakistan (Cigliano et al. 2020).

###### Remarks.

This species was erected by Saeed (2000) from Peshawar, KPK based on a single female specimen; subsequently Malik et al. (2013) reported its male from the Hyderabad -Sindh. We have a single female from the rocky area of Nagarparkar and confirm its presence in the desert area.

##### ﻿ Callogryllus bilineatus

Taxon classificationAnimaliaOrthopteraGryllidae

(Bolívar, 1900)

32BC6687-D252-52D3-A4F2-828FE832B053

Figures 1 , 2 , 3 , 4 , 5 , 6 , 7 , 8 , 9 , 10 , 11 , Table 1

###### Material examined.

Pakistan, **Sindh Prov.** • 2♀; Riffat; 25 Aug. 2019; Islamkot 24.7014°N, 70.1783°E.

###### Description.

Medium size. Colouration brown to yellowish (Fig. 1R). Head brown, short, dome-shaped with four yellowish vertical sutures (Fig. 3G). Pronotum brown, concave anteriorly while pubescent and convex posteriorly with longitudinal rufous bands on dorsal field (Fig. 5H). Elytra scarcely extending to apex of first abdominal tergite, slightly crossing at median line with internal oblique margin, apex rounded; dorsal field plain with straight veins at regular intervals; transverse veinlets very scarce; lateral field with four curved veins (Fig. 10E). Legs yellow, brownish at base, strongly pubescent, irregular bands on dorsal field. Posterior tibiae armed with eleven external, three medio-internal spines (Fig. 7D). Abdomen yellow to dark brown, longitudinal rufous bands on each side. Ovipositor very long, straight, apical valves with dark base (Fig. 1R).

**Female**: LH 3.6 (mm), LP 04 (mm), LT 05 (mm), LF 13.5 (mm), LT 10 (mm), LT 03 (mm), TBL 18 (mm).

###### Ecology.

This species is recorded from wheat crops cultivated at Islamkot, Sindh. Weissman et al. (1980) observed that the hoppers emerged in the early days of June and continued to grow till mid-July. Adults were recorded from then to September. Peak period of species’ occurrence was noted as mid-August to end of September. Thereafter, no individuals were observed in the field. High risk was reported to *Triticum* (wheat) crops from different areas of Islamkot, Sindh (reference).

###### Global distribution.

India, Sindh, Pakistan (Cigliano et al. 2020).

###### Remarks.

Chopard (1969) compiled a detailed account on this species: the head had the same pattern as *C.ovilongus*. The abdomen showed the longitudinal bands on both sides. The elytral length extended from the apex of the abdominal tergite. He calculated body length as 12 mm, pronotum 2.5 mm, elytra 2 mm, and ovipositor 9 mm. The collected specimens show variation in size as well as in other parameters, possibly due to geographical and feeding habitats. This species has unique characteristics, including the presence of a black band that runs from the pronotum where it makes a raised bulging cup-like structure; this black band covers the whole length of tegmen it follows a narrow straight line on the abdominal segments to the end of the last segment.

### ﻿Tribe Modicogryllini

#### Genus *Lepidogryllus* Otte & Alexander, 1983

##### ﻿ Lepidogryllus siamensis

Taxon classificationAnimaliaOrthopteraGryllidae

Chopard, 1961

70C5BD95-6D58-5A0B-AEB1-55796DFEFD1F

Figures 1 , 2 , 3 , 4 , 5 , 6 , 7 , 8 , 9 , 10 , 11 , Table 1

###### Material examined.

Pakistan, **Sindh Prov.** • 1♀; Surriya; 27 Jul. 2019; Ramalani, Umerkot 25.3549°N, 69.7376°E.

###### Description.

Medium size. Colouration dark brown (Fig. 1S). Head shiny brown, short, narrow, ocelli black, horizontal dark band between (Fig. 3H). Pronotum as long as head, 2 × wider than long on dorsal field, anterior and posterior margin pilose, truncated, dorsal surface brownish, mottled; lateral lobe of pronotum a little deeper than pronotal length (Fig. 5I). Elytra hardly reaching abdominal end. Wings well developed, with condensed veins (Fig. 10F). Legs brown, hind femora much longer than middle femora. Posterior tibia armed with seven external, three medio-internal spines, very wide at anterior, numerous patches on dorsal surface (Fig. 7E). Abdomen brown. Cerci long tapered. Ovipositor long, straight, with yellowish base (Fig. 1S).

**Female**: LH 1.96(mm), LP 2.03(mm), LT 9.5(mm), LF 5.6(mm), LT 07(mm), LT 04(mm), TBL 11(mm).

###### Ecology.

This species was recorded for the first time from the village Ramalani, Umerkot, on the roots of *Acacianilotia* locally known as “babul”. This is a medium-sized, thorny, nearly evergreen tree found in the desert area. Generally, it grows to 20–25 mm but may remain shrubby in poor conditions. Our specimen was collected from a shrub. This tree provides limber, fuel, shade, food, dye, and gum, and it also impacts the environment positively through soil reclamation.

###### Global distribution.

Korea, Japan, Taiwan, Thailand, India, Hawaii, China, Pakistan (Cigliano et al. 2020).

###### Remarks.

*Lepidogryllus* has a very close morphological resemblance with *Velarifictorus*: the male has an enlarged round head with a swollen frons (Randell, 1964). Kim (2013) also reported the many similarities between these two genera. The species of these genera also have very significant variation in their morphometric parameters. Kim (2013) reported a body length of 14–15.2 mm in *L.siamensis*; we report a body length 11 mm.

### ﻿Oecanthinae

**Oecanthini**

#### Genus *Oecanthus* Serville, 1831

##### ﻿ Oecanthus fultoni

Taxon classificationAnimaliaOrthopteraGryllidae

Walker, 1962

32615D68-E203-5BE0-83E2-80AD459A94C8

Figures 1 , 2 , 3 , 4 , 5 , 6 , 7 , 8 , 9 , 10 , 11 , Table 1

###### Material examined.

Pakistan, **Sindh Prov.** • 1♀; Riffat; 16 Aug. 2020; Umerkot 25.3549°N, 69.7376°E.

###### Description.

Large size. Colouration light pale green to yellowish (Fig. 1T). Head short, narrow with dark brown ocelli (Fig. 3I). Pronotum flat, concave posteriorly (Fig. 5J). Elytra, transparent, extending to 2/3 of abdomen. Wings rounded, broad, with condensed irregular veins (Fig. 10G). Legs same colour as body. Femora long, thin, slightly wider at anterior and compressed at posterior. Posterior tibia thin, slender, armed with 21 external and three medio-internal spines (Fig. 7F). Abdomen pale yellowish. Ovipositor short. Cerci long with pointed ends (Fig. 1T).

**Female**: LH 1.96 (mm), LP 2.73 (mm), LT 14 (mm), LF 3.57 (mm), LT 3.85 (mm), TBL 22 (mm).

###### Ecology.

*Oecanthusfultoni* is a new record from Umerkot, Desert Thar, Pakistan. This species is reported from *Cynadondactylon* (common lawn grasses) surrounded by wild plants.

###### Global distribution.

Ohio, Franklin, New Jersey, Washington, Pakistan (Cigliano et al. 2020).

###### Remarks.

Walker and Gurney (1967) observed differences between populations of this species from the coasts of western and eastern USA showing that *O.fultoni* displays variations in the structure of the metanotal gland.

### ﻿Key to the genera of *Gryllidae* of Sindh

**Table:** 

1	Head brown with two variables extending testaceous bands (Fig. 2A, B). Pronotum adorned with two large brown bands (Fig. 4A, B)	**2**
_	Head curved feebly at anterior; wider at posterior (Fig. 1D, E). Pronotum concave with piriform impression on anterior disc (Fig. 4D, E)	**3**
2	Elytra extending to the apex of abdomen, mirror small, obliquely transverse (Fig. 8C). Wings long. Legs pale yellowish with numerous hairs. Tibia with eleven pointed spines on either side (Fig. 6C). Abdomen yellow, pubescent. Cerci well developed, pointed	***Acheta* Linnaeus**
_	Elytra extending to 1/3 of abdominal tergite, truncated, rounded at apex; mirror quite apical, little wider than long, rounded posteriorly; wings reduced (Fig. 9B). Legs pubescent, yellowish, with few brown spots. Anterior tibia perforated on the external face with a rather long, oval tympanum (Fig. 6H). Abdomen brown with triangular median line on dorsal field	**4**
3	Legs blackish testaceous with brown spots, pubescent. Posterior femora rather short and thick; posterior tibia armed with six spines on each margin (Fig. 1F). Abdomen brown, ovipositor long, slender with narrow, very acute apical valves (Fig. 1F)	***Gryllus* Linnaeus**
_	Legs brownish, fuscous; posterior femora moderately swollen, striated on external face; posterior tibiae armed with seven spines on each margin (Fig. 6I). Abdomen pale brown, yellowish beneath. Ovipositor long, slender (Fig. 1J)	**5**
4	Head small, narrow at the anterior, slightly curved at posterior. Face short, yellow with spotted clypeus. Frontal suture feebly arched (Fig. 2I). Pronotum transverse, feebly concave at anterior (Fig. 4I)	***Gryllodes* Saussure**
_	Head short, yellow, adorned with rufous spots, dorsal field of ocelli with pubescent horizontal dark bands (Fig. 3B). Pronotum depressed above with straight yellowish posterior margin on dorsal field (Fig. 5C)	**6**
5	Colour pale brown (Fig. 1J). Head brown to dark with horizontal band at posterior margin. Ocelli dark brown. Pronotum dark brown, enlarged in front, its surface is rather strongly punctuated with numerous testaceous rufous spots (Fig. 4J)	***Teleogryllus* Chopard**
_	Colour rufous brown, shiny (Fig. 1N). Head little wider than pronotum in front; occiput convex with frontal rostrum narrow, ocelli united by a small oblique keel (Fig. 3C). Pronotum dark brown, slightly broader than long with concave anterior margin, posterior margin feebly convex (Fig. 5D)	**7**
6	Legs brownish. Pubescence rather thick, compressed. Anterior tibia bearing small, oval, external tympanum. Posterior tibia armed with ten external and one medio-internal spines (Fig. 6L). Abdomen brown. Ovipositor short, straight, slender with very small, lanceolate, acute apical valves (Fig. 1M)	***Modicogryllus* Chopard**
_	Legs dark brown dorsally, whitish ventrally. Tibia armed with nine internal, four medio-internal spines (Fig. 7B). Abdomen black, sternites whitish. Ovipositor long, slender, straight with lanceolate apical valves (Fig. 10)	**8**
7	Elytra extending to the apex of abdomen, narrow posteriorly. Wing well developed (Fig. 10A). Ovipositor rather long, straight with lanceolate apical valves. Abdomen brown. (Fig. 1N)	***Svercus* Gorochov**
_	Elytra scarcely extending to apex of first abdominal tergite, slightly crossing at median line with internal oblique margin, (Fig. 10E). No wings. Ovipositor very long, straight, apical valves with dark base. Abdomen yellow to dark brown, longitudinal rufous bands on each side. (Fig. 1R)	**9**
8	Colour brown (Fig. 10). Head black bright and globous; whitish spot posteriorly containing scape and following inner margins of eyes, becoming punctuated with brown with white stripe before reaching occiput (Fig. 3D). Pronotum black with pubescence, dorsal disc wider than long, bristles on anterior and posterior margins; lateral lobes marked with antero-ventral whitish spot which becomes pale brown posteriorly (Fig. 5E)	***Miogryllus* Saussure**
_	Colour dark brown (Fig. 1S). Head shiny brown, short, narrow, ocelli black, horizontal dark band between (Fig. 3H). Pronotum as long as head, 2 × wider than long on dorsal field, anterior and posterior margin pilose, truncated, dorsal surface brownish, mottled; lateral lobe of pronotum a little deeper than pronotal length (Fig. 5I)	***Lepidogryllus* Otte & Alexander**
9	Head brown, short, dome-shaped with four yellowish vertical sutures (Fig. 3G). Pronotum brown, concave anteriorly while pubescent and convex posteriorly with longitudinal rufous bands on dorsal field (Fig. 5H). Legs yellow, brownish at base, strongly pubescent, irregular bands on dorsal field. Posterior tibiae armed with eleven external, three medio-internal spines (Fig. 7D)	***Callogryllus* Sjöstedt**
_	Head pale green, narrow with dark brown ocelli (Fig. 3I). Pronotum flat, concave posteriorly (Fig. 5J). Legs same colour as body. Femora long, thin, slightly wider at anterior and compressed at posterior. Posterior tibia thin, slender, armed with 21 external and three medio-internal spines (Fig. 7F)	***Oecanthus***

### ﻿Serville Keys to the species of *Gryllidae* of Sindh

**Table:** 

1	Body colouration pale brown, fulvous, or testaceous (Fig. 1A). Head brown with 2 variably extending testaceous bands (Fig. 2A, B). Pronotum with 2 large brown spots (Fig. 4A, B). Posterior tibia armed with 11 spines on the basal side (Fig. 6A, B)	***Achetadomesticus* Linnaeus**
_	Body colouration brownish yellow, rather large, robust (Fig. 1C). Head blackish with shiny occiput (Fig. 2C). Pronotum unicolourous, concave, very slightly widening anteriorly and posteriorly; posterior margins with numerous spots, without large brown spots, double line anteriorly and posteriorly (Fig. 1C). Tibia with 1 pointed spine on either side (Fig. 6C)	**2**
2	Elytra extending to the apex of abdomen (Fig. 8C). Wings long. Legs pale yellowish with numerous hairs (Fig. 6C)	**3**
_	Elytra reach to the top of abdomen, wings much long (Fig. 8D, E). Legs dark brown, strongly pubescent (Fig. 1D, E)	**4**
3	Abdomen yellow, pubescent; ovipositor long, straight, serrated with numerous sutures. Cerci well developed, pointed at the terminus (Fig. 1C)	***A.hispanicus* Rambur**
_	Abdomen brown; ovipositor moderately long, rather slender with apical valves very acute (Fig. 1G). Cerci small, tapered at apex	**6**
4	Body large, stout. Colour blackish. Head curved feebly anteriorly; wider at posterior (Fig. 1D, E). Pronotum concave with piriform impression on anterior disc (Fig. 4D, E)	**G. (Gryllus) bimaculatus De Geer**
_	Body size medium to large. Colour brown. Head yellowish brown with patches and raised veins (Fig. 2F). Pronotum convex above, blackish brown with fine greyish pubescens; posterior margin sinuated (Fig. 4F)	**5**
5	Elytra run beyond length of body, elytra with yellow patches on base	***G.campestris* Linnaeus**
_	Elytra equilateral reduced, extending to the extremity of abdomen, wings caudate (Fig. 9C)	**7**
6	Fastigium of vertex black, shiny, flat, slightly curved at sides, large body size, elytra large with thick venation system along total body length	***G.septentrionalis* F. Walker**
_	Fastigium of vertex yellowish brown, curved at the anterior side; body elongated, elytra small, disjointed	***Gryllodessigillatus* Walker**
7	Head small, brown, with narrow frontal rostrum, pronotum transverse, feebly concave anterior margin; elytra of female are moderately diverse	**8**
_	Head wide at back and narrow in front, pronotum concave and slightly broad, Face blackish brown, right wing overlappintg on anterior wing	**T. (Brachyteleogryllus) occipitalis Serville**
8	Femur thick at anterior but narrow at posterior, without spines. Tibia moderately thin, armed with 10 anterior spines, no spines on external side	***G.supplicans* Walker**
_	Femur thick, small, banded with vertical lines. Tibia thin with pointed spines with black bases, dorsal field of tegmina with several veins	**9**
9	Head short with vertical light and dark bands at posterior margin. Ocelli dorsal field with dark horizontal band (Figs 2J, 3A). Pronotum dark brown, variably fulvous with black inferior margin (Fig. 5A, B)	.**T. (Brachyteleogryllus) commodus Walker**
_	Head larger, yellow, adorned with rufous spots, ocelli dorsal field with dark, horizontal, pubescent bands (Fig. 3B). Pronotum depressed above with straight yellowish posterior margin; dorsal field coarse (Fig. 5C)	**10**
10	Femur wide with numerous patches and immovable spines, tibia has several spines on one side, tegmina dorsal field with 3 or 4 oblique veins	**11**
_	Femur thick, small groove at anterior, small hairs on external and internal sides. Tibia armed with 9 external, 11 internal, and 2 medio-internal spines	***Svercuspalmetorum* Krauss**
11	Elytra extending to the apex of abdomen; veins of the dorsal field rather irregular and condensed (Fig. 9G)	**12**
_	Elytra extending to 2/3 of the abdomen, apical field well developed; wings surpassing abdomen tip (Fig. 10B)	**13**
12	Abdomen brown. Ovipositor short, straight, slender with very small lanceolate, acute apical valves (Fig. 1M)	***Modicogryllussindhensis* sp. nov.**
_	Abdomen yellow with dark spots on each tergite. Ovipositor long, straight, slender (Fig. 1P)	**14**
13	Legs dark brown dorsally, whitish ventrally. Tibia armed with 9 internal and 4 medio-internal spines (Fig. 7B). Abdomen black, sternites whitish. Cerci pale brown, short. Ovipositor long, slender, straight with lanceolate apical valves (Fig. 1O)	***Miogryllusitaquiensis* Orsini & Zefa**
_	Legs brown, hind femora much longer than middle femora. Posterior tibia armed with 7 external, 3 medio-internal spines, much wider anteriorly, numerous patches on dorsal surface (Fig. 7E). Abdomen brown. Cerci long, tapered. Ovipositor long, straight with yellowish base (Fig. 1S)	1**5**
14	Medium size. Colouration yellow (Fig. 1P). Head short, narrow, yellowish, shiny, adorned on each side with dark brown line extending from occiput, along eye (Fig. 3E). Pronotum as long as wide, feebly widening in front with two dark spots on dorsal field (Fig. 5F). Elytra reduced. No wings (Fig. 10C). Ovipositor slim and acute	***Callogryllussaeedi* Saeed**
_	Medium size. Colouration yellow (Fig. 1Q). Head short, narrow, very neat. Eyes rounded, moderately projecting; ocelli small (Fig. 3F). Pronotum 1.5 × as wide as long, slightly concave at anterior margin, straight posteriorly, one side rather strongly convex (Fig. 5G). Elytra yellow, small (Fig. 10D). With or without wings. Ovipositor small, very elongated, acute slim apical valve	***C.ovilongus* Saeed & Yousuf**
15	Eyes oval and brown, pronotum serrated overall and wide, abdominal part smaller than tegmen, wings large. Legs yellow, brownish at base, strongly pubescent, irregular bands on dorsal field. Posterior tibiae armed with 11 external and 3 medio-internal spines (Fig. 7D)	***C.bilineatus* Bolívar**
_	Eyes small, oval, bulging outwards, ocelli black, horizontal dark band present (Fig. 3H). Pronotum as long as head, dorsal field 2 × wider than long, anterior and posterior margins pilose, truncated, dorsal surface brownish, mottled (Fig. 5I). Wings with condensed veins (Fig. 10F). Legs brown, hind femora much longer than middle femora. Posterior tibia armed with 7 external, 3 medio-internal spines (Fig. 7E)	**16**
16	Fastigium of vertex circular, brownish, shiny. Eyes small, dark brown. Head shiny brown, short, narrow; ocelli black, with horizontal dark band (Fig. 3H). Tegmen pointed at one end and curved at the other. Tibia with 10 spines. Cerci long tapered. Ovipositor long, straight, with yellowish base (Fig. 1S)	***Lepidogryllussiamensis* (Chopard)**
_	Fastigium of vertex small, tapered, green. Eyes black. Head short, narrow with dark brown ocelli (Fig. 3I). Tegmen snowy transparent extending to 2/3 tip of abdomen. Wings rounded, broad with condensed irregular veins (Fig. 10G). Tibia thin, slender armed with 21 external, 1 medio-internal spines (Fig. 7F). Cerci long with pointed ends. Ovipositor short (Fig. 1T)	***Oecanthusfultoni* Walker**

## Supplementary Material

XML Treatment for Acheta domesticus

XML Treatment for Acheta hispanicus

XML Treatment for Gryllus (Gryllus) bimaculatus

XML Treatment for Gryllus (Gryllus) campestris

XML Treatment for Gryllus septentrionalis

XML Treatment for ﻿Gryllodes sigillatus

XML Treatment for Gryllodes supplicans

XML Treatment for Teleogryllus (Brachyteleogryllus) occipitalis

XML Treatment for Teleogryllus (Brachyteleogryllus) commodus

XML Treatment for Modicogryllus sindhensis

XML Treatment for Svercus palmetorum

XML Treatment for Miogryllus itaquiensis

XML Treatment for Callogryllus saeedi

XML Treatment for Callogryllus ovilongus

XML Treatment for Callogryllus bilineatus

XML Treatment for Lepidogryllus siamensis

XML Treatment for Oecanthus fultoni
